# Modelling the productivity of Siberian larch forests from Landsat NDVI time series in fragmented forest stands of the Mongolian forest-steppe

**DOI:** 10.1007/s10661-021-08996-1

**Published:** 2021-03-18

**Authors:** Stefan Erasmi, Michael Klinge, Choimaa Dulamsuren, Florian Schneider, Markus Hauck

**Affiliations:** 1 Thuenen Institute of Farm Economics, Bundesallee 63, 38116 Braunschweig, Germany; 2 Department of Physical Geography, Institute of Geography, University of Goettingen, Goldschmidtstraße 5, 37077 Goettingen, Germany; 3 Applied Vegetation Ecology, Faculty of Environment and Natural Resources, University of Freiburg, Tennenbacher Str. 4, 79106 Freiburg, Germany

**Keywords:** Forest-steppe, NDVI, Tree productivity, Tree ring, Remote sensing, Dendrochronology

## Abstract

The monitoring of the spatial and temporal dynamics of vegetation productivity is important in the context of carbon sequestration by terrestrial ecosystems from the atmosphere. The accessibility of the full archive of medium-resolution earth observation data for multiple decades dramatically improved the potential of remote sensing to support global climate change and terrestrial carbon cycle studies. We investigated a dense time series of multi-sensor Landsat Normalized Difference Vegetation Index (NDVI) data at the southern fringe of the boreal forests in the Mongolian forest-steppe with regard to the ability to capture the annual variability in radial stemwood increment and thus forest productivity. Forest productivity was assessed from dendrochronological series of Siberian larch (*Larix sibirica*) from 15 plots in forest patches of different ages and stand sizes. The results revealed a strong correlation between the maximum growing season NDVI of forest sites and tree ring width over an observation period of 20 years. This relationship was independent of the forest stand size and of the landscape’s forest-to-grassland ratio. We conclude from the consistent findings of our case study that the maximum growing season NDVI can be used for retrospective modelling of forest productivity over larger areas. The usefulness of grassland NDVI as a proxy for forest NDVI to monitor forest productivity in semi-arid areas could only partially be confirmed. Spatial and temporal inconsistencies between forest and grassland NDVI are a consequence of different physiological and ecological vegetation properties. Due to coarse spatial resolution of available satellite data, previous studies were not able to account for small-scaled land-cover patches like fragmented forest in the forest-steppe. Landsat satellite-time series were able to separate those effects and thus may contribute to a better understanding of the impact of global climate change on natural ecosystems.

## Introduction

Terrestrial carbon of aboveground biomass plays a major role in the global carbon cycle. Knowledge about productivity and spatial distribution of aboveground carbon stocks in forests is essential for global biogeochemical scenarios and modelling (Goodale et al., 2002; Pan et al., 2011). Assessing the spatial and temporal variability of vegetation productivity remains a challenge and is a function of numerous environmental factors. Changing climate is assumed to have a considerable impact on vegetation growth and hence, tree productivity, especially in areas that are considered as hotspots of global climate change, like the eastern part of Central Asia (Turco et al., 2015). Direct measurements of tree productivity on historical scales are available from dendrochronological studies that use the annual radial stem increment (tree ring width; TRW) as a measure of tree growth and thus are able to produce time series of ecosystem productivity. Depending on the climatic conditions, annual stem increment in boreal of forest can either be limited by low temperatures or drought during the growing season (Beck et al., 2011; Hauck et al., 2019; Zhou et al., 2020). As the result of climate warming, boreal forests are increasingly switching from temperature-limited to drought-limited ecosystems (D'Arrigo et al., 2004; Buermann et al., 2014). Especially the forest-steppe related to the semi-arid regions of Central Asia is more and more subjected to forest disturbance by fire and windthrow (Nyamjav et al., 2007). These forests often show decreases in productivity (Dulamsuren et al., 2010, 2013).

Tree ring studies can cover centuries but only deliver local information. The uneven and sparse distribution of available tree ring information cannot represent larger areas. By contrast, remote sensing data are able to monitor and quantify ecological parameters that affect plant productivity over large areas. The basic concept relies on the establishment of relations between the absorbed and reflected solar radiation in the visible and near infrared (VNIR) spectrum and the specific index of plant productivity in question, such as tree ring increment (Eitel et al., 2020). The vast majority of such studies use regression analyses to parameterize the relationship between the target variable and the remote sensing observations (Lu et al., 2015; Powell et al., 2010; Rodríguez-Veiga et al., 2019). In most cases, when long time series of satellite data are investigated, the Normalized Difference Vegetation Index (NDVI) serves as a proxy variable (Eckert et al., 2015; Fensholt & Proud, 2012; Ivanova et al., 2021; Testa et al., 2018). The NDVI is calculated as the ratio between the difference and the sum of the spectral reflectance in the red and near-infrared region (Rouse et al., 1974).

Combining temporal series of ground-measured tree-ring and remote-sensing data enables the generation of a data pool for the assessment of the spatial and temporal variability in forest productivity for the last four decades since operational satellite earth observations became available. Several studies exist that have demonstrated the potential of satellite remote sensing time series for tree productivity monitoring. Bunn et al. (2013) used time series of bi-weekly NDVI data for the estimation of tree growth and revealed best correlations for July NDVI data together with the first principal component of tree-ring data from different sites of the Siberian taiga. Comparable results were reported from juniper woodlands of the Tibetan Plateau by He and Shao (2006) who also used the first principal component of the tree-ring data together with NDVI time series. Kaufmann et al. (2004) and Xu et al. (2019) underlined the importance of the summer greenness of the canopy (June/July) for the prediction of TRW. D'Arrigo et al. (2000) and Wang et al. (2004) also highlighted the potential of satellite-time series for the prediction of forest productivity. However, not all studies confirmed a general positive relationship between dendrochronological sampling and annual or seasonal NDVI composites. A recent study by Brehaut and Danby pointed out that relations are inconsistent and might be a function of forest type and also be affected by climatic variables (Brehaut & Danby, 2018).

All mentioned studies have in common that they were based on data from coarse spatial resolution sensors (e.g. AVHRR, MODIS) and cannot account for small-scaled land-cover patches and landscape heterogeneity. Due to this limitation in resolution and the highly fragmented forests in Central Asia, some studies used NDVI time series over larger grassland areas as a proxy for forest productivity (He & Shao, 2006). Only few and recent studies made use of the potential of higher spatial resolution time series available from the Landsat missions for mapping spatial and temporal variations in forest productivity (Liu, 2016). Coops et al. (1999) was the first to evaluate the potential of annual Landsat data stacks for the estimation of the annual increment of forest-stand volume. Powell et al. (2010) compared different regression techniques to estimate biomass from time series of Landsat data. Main-Knorn et al. (2013) and Thomas et al. (2011) monitored forest disturbance and regrowth patterns from biennial time series stacks. The only study that used a multi-year series of Landsat for the estimation of tree ring width so far was published by Sangüesa-Barreda et al. (2014). They used single Landsat-5 images for 15 irregularly distributed years within a 25-year span to monitor the consequences of insect outbreaks on the annual increment of the stand basal area in Mediterranean pine forests. A major issue with such approaches is the proper determination of a consistent time of the year when dealing with long annual time series. All known studies that are related to predicting forest productivity from Landsat time series only manually selected the optimal annual acquisition based on quality criteria (cloud coverage, peak of growing season) and only made use of a single sensor.

In the present study, we investigated the applicability of Landsat-based growing season NDVI time series as a proxy of TRW of Siberian larch (*Larix sibirica*) at the southern border of the boreal forests in Mongolia. Landsat data covered a range of 32 years from 1986 to 2017. In addition to existing studies, we used data from a full dense Landsat time series (LTS) of all available acquisitions from the Landsat-sensors Landsat-5/TM, Landsat-7/ETM+, and Landsat-8/OLI. We computed annual-monthly metrics of NDVI data for the growing season over all sensors as a proxy for tree productivity. Besides the evaluation of the technical potential of the LTS, the central hypothesis of the study was that vegetation greenness is a function of tree ring width, and hence, NDVI LTS are able to capture temporal anomalies and trends in forest productivity due to climate variations. Based on the findings of Khansaritoreh et al. (2017) that the forest-steppe occurs in a dominantly drought-limited environment, our main hypothesis referred to the general assumption that long time series of high-resolution satellite data indicate the temporal variations in vegetation greenness that are related to climate variations. Furthermore, we postulated that the spatial patterns of those trends are related to forest fragmentation, i.e. that the reduction of forest areas (i.e. the forest-to-grassland ratio) has an impact on the vulnerability to climate variations and thus tree productivity. Further, it was tested, if steppe-grassland areas surrounding the forest stands can serve as a proxy for the monitoring of tree growth when the resolution of the satellite data and the size of the forest patches is not large enough for the analysis of homogeneous areas.

## Material and methods

**Fig. 1:**
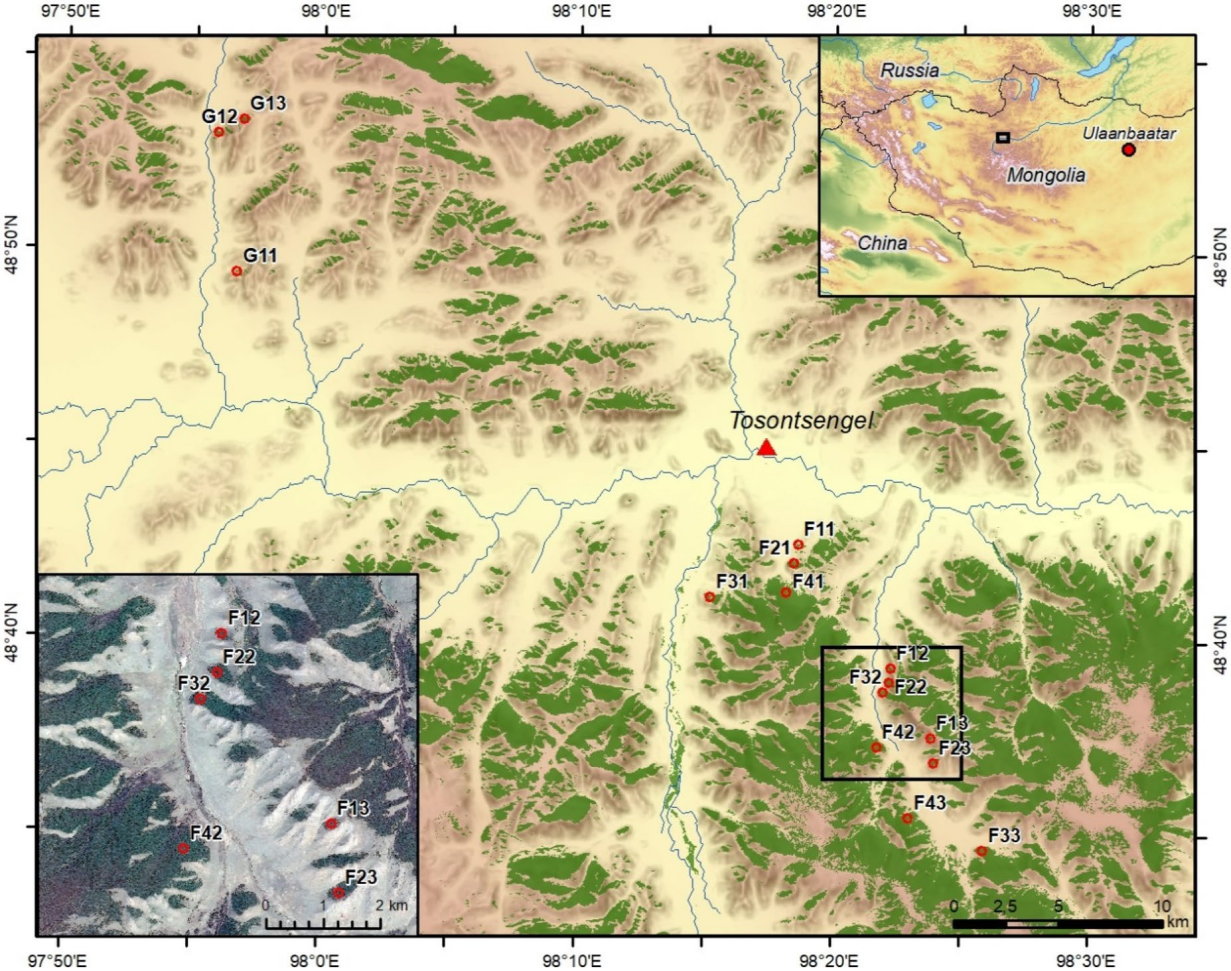
Study area near Tosontsengel, Mongolia with clusters of forest stands of different size (increasing from F1/G1 to F4) in subregions with high (F1–F4, south-eastern part of the study area) or low (G1, north-western part) forest-to-grassland ratio. The red points indicate the plot positions and the first digit defines the patch size class, whereas the second digit numbers the replica. The small rectangle in the overview map (top right) shows the location of the study area (main map). The rectangle in the main map shows the position of the detailed map (bottom left)

**Fig. 2:**
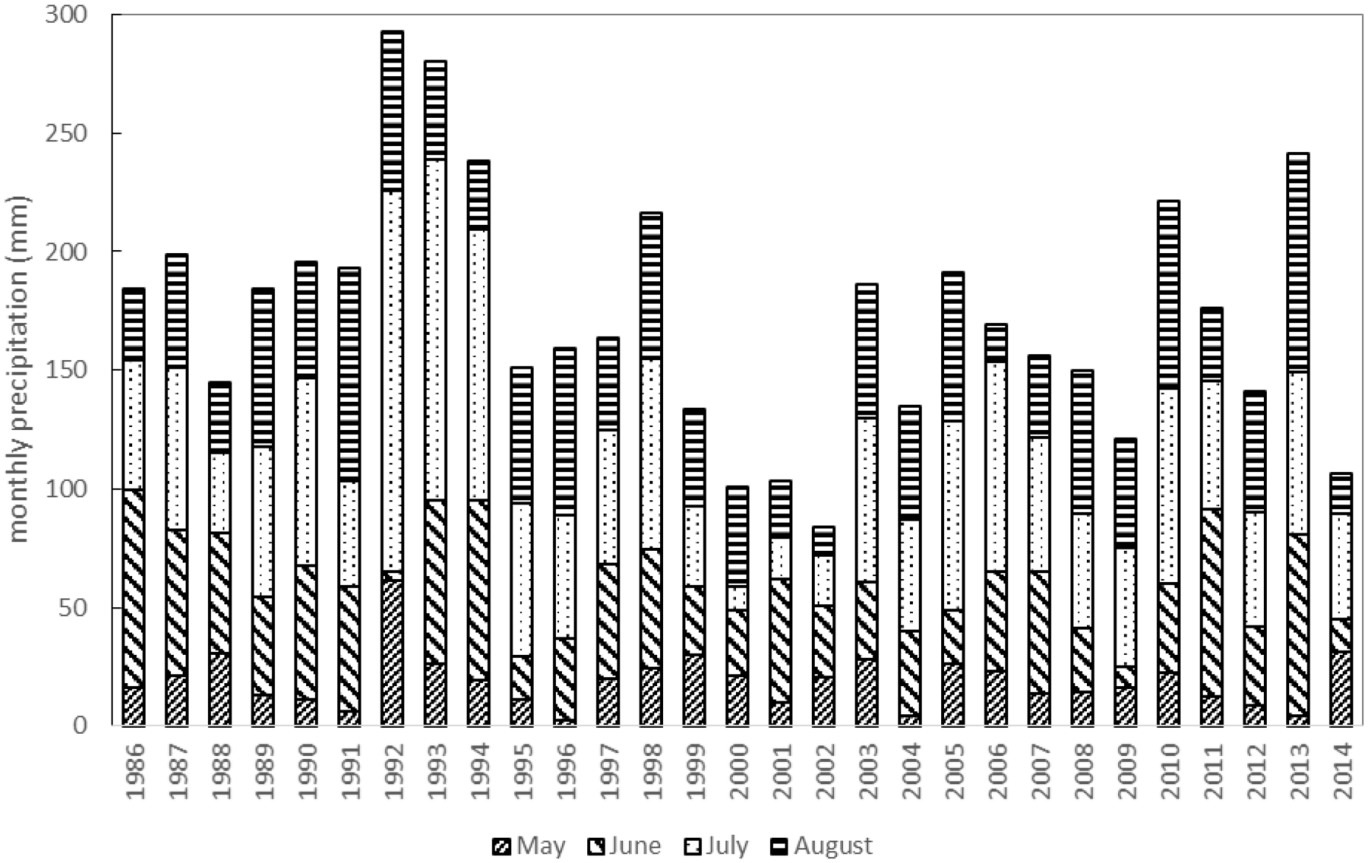
Variation of monthly precipitation during the summertime in the study area from 1986 to 2014 (daily data for Tosontsengel, aggregated to monthly sums and cumulated for the period May to August). Climate data were obtained from the National Agency for Meteorology and Environment Monitoring of Mongolia, Ulan Bator

The study area is located at the northern slope of the Khangai Mountains near the city of Tosontsengel in central Mongolia (98°16′E/48°46′N) about 600 km to the west of Ulan Bator (Fig. 1). The high continental climate of the region is reflected by cold and semi-arid conditions. The monthly mean temperatures at Tosontsengel range between − 31.7 °C in January and 14.7 °C in July (National Agency for Meteorology and Environment Monitoring of Mongolia, Ulan Bator). The main precipitation occurs during the summer season and is related to the circulation of the westerlies (Batima et al., 2005). Summer precipitation is subject to high inter-annual variations (Fig. 2), making the area vulnerable to droughts. In contrast, the Siberian High during the winter season produces mostly dry conditions. The area is part of the southern boreal forests of Mongolia with altitudes ranging from 1600 to 2500 m a.s.l. Forest stands are strongly dominated by Siberian larch (*Larix sibirica*) and occur mainly on northern slopes whereas grassland steppe is found on the southern slopes (Hilbig, 1995; Treter, 1996). The major factors limiting tree growth are the shortage of precipitation (< 300 mm/a) together with high evapotranspiration as a function of incoming solar radiation and terrain parameters (Hais et al., 2016; Schlütz et al., 2008). Forest stands in the study area show considerable impact from industrial timber harvest activities in the second half of the twentieth century until 1990. After the end of the socialistic period in Mongolia, activities changed to predominantly unsystematic selective logging by the rural population (Nyamjav et al., 2007). Today, the forest-steppe area is home to mobile pastoralists, who keep mixed herds of sheep, goats, cattle, yak, and horses on common pastures. Livestock is not much herded and animals preferentially graze on grassland, but also move into the forests along the edges and further into the interior, when the forest islands are small (Lkhagvadorj et al., 2013).

## Field data

Field data of wood cores were taken in August 2014 in monospecific *L. sibirica* forest stands in sub-plots of 20 × 20 m size (Khansaritoreh et al., 2017). Wood cores from all *L. sibirica* trees (1755 individuals) with a stem diameter of ≥ 3 cm were collected at breast height (1.3 m above the ground) using an increment borer with an inner diameter of 5 mm. From the wood cores, the tree ring width was measured to an accuracy of 10 µm using a Lintab 5 measuring system (Rinntech, Heidelberg, Germany). All sampled trees were classified into four age classes including “very old trees” (> 160 years), “old trees” (101–160 years), “middle-aged trees” (61–100 years) and “young trees” (≤ 60 years). The forest stands were selected by a stratified random sampling approach in the core areas of forests (i.e. avoiding a 30 m forest edge buffer). Stratification was based on a supervised classification between forest and non-forest area (Landsat 8 OLT/TIRS of September 19, 2013). All forest patches were subsequently classified into four different size classes (1: < 0.1 km^2^; 2: 0.1–1.0 km^2^; 3: > 1.0–5.0 km^2^; 4: > 5.0 km^2^) that serve as a proxy for forest fragmentation. Based on the forest-to-grassland ratio, we distinguished between grassland-dominated (G) and forest-dominated (F) sub-regions. Forest patches in the grassland-dominated subregion mostly consisted of the smallest patch size class (G1). For each patch class (F1 to F4; G1), three replica were sampled. In each forest stand, two independent nearby sub-plots of 20 × 20 m were sampled. For this study, the data from the sub-plots were aggregated at the stand level. This yielded a total of 15 forest plots distributed over the study area (Fig. 1) that were investigated for the impact of patch size and isolation on the forest productivity.

## Satellite data

Landsat data were collected from U.S. Geological Survey (USGS) Earth Resources Observation and Science (EROS) Center from the Landsat sensors Thematic Mapper (TM), Enhanced Thematic Mapper + (ETM+), and Operational Land Imager (OLI) at a 30-m spatial resolution (U.S. Geological Survey, Earth Resources Observation and Science Center, 2012a, 2012b; U.S. Geological Survey, Earth Resources Observation Science Center, 2014). The “Collection 1 Level-2” scenes are processed to surface reflectance. Landsat-8 OLI surface reflectance data are computed using the Landsat Surface Reflectance Code (LaSRC). Landsat 5 TM and Landsat 7 ETM+ surface reflectance are generated using the Landsat Ecosystem Disturbance Adaptive Processing System (LEDAPS) algorithm. Both algorithms make use of the Moderate Resolution Imaging Spectroradiometer (MODIS) for the estimation of climate and atmospheric parameters but build on two different radiative transfer models to retrieve surface reflectance. LaSRC in addition uses the coastal aerosol spectral band from Landsat-8 OLI to perform aerosol inversion as input for the radiative transfer modelling. Details about the processing of the surface reflectance for the three sensors can be found in the respective product guides (U.S. Geological Survey, 2019a, 2019b). The output from the two processing systems is a compilation of all spectral bands in the solar reflectance domain (Bands 1–5 and 7 for Landsat 5 TM and Landsat 7 ETM+; Bands 2–7, 9 for Landsat 8 OLI) together with a pixel quality assessment layer (“pixel_qa”) that stores a bit index that later is used for cloud masking in the LTS processing. It should be noted that, depending on the surface reflectance processing algorithm, this bit index stores different values for clear pixels. On demand, the processor also delivers a collection of ready to use vegetation indices like the NDVI and others.

**Fig. 3:**
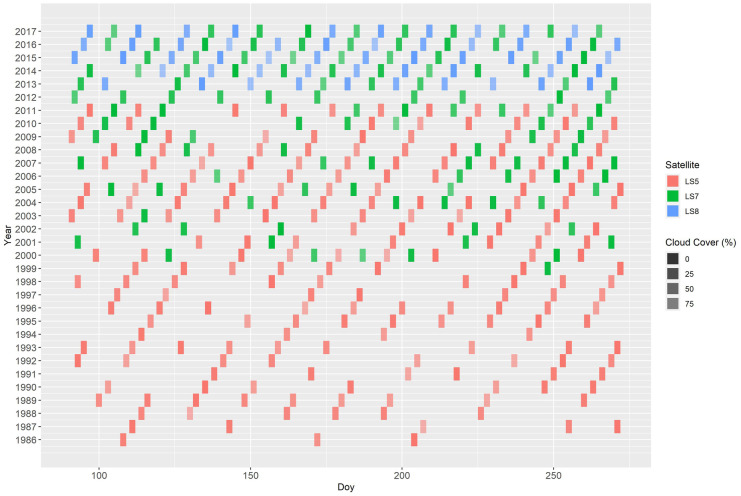
Summary of all available Landsat satellite imagery for the study area between 1986 and 2017 (Landsat path 137/row 26; colour of the bars shows the three Landsat systems, red = Landsat-5 TM, green = Landsat 7 ETM+, blue = Landsat 8 OLI; opacity of the bars indicates amount of cloud coverage)

For the purpose of time-series analysis, only highest quality data with a root mean square error of less than or equal to 12 m in geometric accuracy (referred to as “Tier 1” Landsat data) were used in the present study according to the recommendations of the U.S. Geological Survey (U.S. Geological Survey, 2019c). All Landsat scenes (path 137/row 26) from beginning of April until end of September were selected for the 31-year time period 1986 to 2017, from which at least a subset of 20 years was used (see below). This resulted in an overall collection of 216 scenes from the TM sensor, 123 from ETM+, and 55 from OLI. An overview of all Landsat data for this study is given in Fig. 3.

## Data analysis

The collection of Landsat satellite data for the study area was processed to a time series layer stack of NDVI images. First, the NDVI was computed for every single Landsat scene based on the well-known ratio of high absorption in the Red and high reflectance of incoming solar radiation in the NIR part of the electromagnetic spectrum as an indicator for “green” vegetation and vegetation productivity (Tucker, 1979):

$$NDVI=\frac{NIR-Red}{NIR+Red}$$

Cloud, cloud-shadow, snow, and ice-affected pixels were masked for each NDVI layer based on the pixel quality assessment layer of the respective Landsat acquisition. Differences in reflectance wavelength and hence, NDVI, between the OLI and the other two sensors have been observed for stable land surface targets. These differences are a result of the improved calibration, radiometric, and spectral resolution of the OLI sensor. In order to produce a consistent LTS, we accounted for this issue based on ordinary least square (OLS) regression transformations of the NDVI from Landsat 8 OLI to Landsat 7 ETM+ and Landsat 5 TM that are suggested by Roy et al. (2016).

After masking and transformation, the Landsat NDVI data were aggregated using a maximum value compositing (MVC) approach (Holben, 1986) for all data that were acquired within the long-term growing season between mid of May and mid of September for every single year. The MVC approach selects for every pixel the maximum NDVI value from a satellite dataset recorded during the growing season of 1 year. Therefore, these aggregated annual values are referred to as the “maximum growing season NDVI (MGS-NDVI)” in this study. Data availability within the compositing period may produce a bias in the MGS-NDVI when only few images are available. Further, strong rainfall during the summer months around the peak of growing season may introduce artefacts in the composited seasonal NDVI time series. E.g., during 1992 and 1994, climate records show extreme precipitation sums during the summer months (especially in July, see Fig. 2). These issues restricted the processing of the MGS-NDVI to a minimum of three cloud free observations (at the pixel level) per season. As a consequence, considerable parts of the study area were masked out, especially for the first 10 years of the Landsat collection where only Landsat-5 images are available and at irregular temporal intervals with a considerable lack of data at the peak of the growing season where NDVI is at its maximum (Fig. 3). Thus, the final time series included only the years from 1995 to 2017. With regard to the availability of the tree ring data, only the period from 1995 to 2014 could be evaluated.

The two-time series of annual data (TRW and MGS-NDVI) from 1995 to 2014 were finally corrected for seasonal effects and adjusted to a comparable data range by computing the standardized anomalies:

$$MGSNDVI_a=\frac{MGS\;NDVI-\mu_{MGSNDVI}}{\sigma_{MGSNDVI}}$$

where *μ*_MGS-NDVI_ is the mean value of the MGS-NDVI observations in the time period 1995 to 2014 and *σ*_MGS-NDVI_ is its standard deviation. The standardized anomalies of TRW are calculated as TRW_a_, respectively.

The analysis concept of the relations between MGS-NDVI_a_ and TRW_a_ built on a correlation analysis at the level of the sub-categories (F1-F4; G1) that are defined above. The relationships were investigated for every single forest plot (with *n* = no. of available years) and for the mean values of the standardized anomalies across the three forest plots within each patch size category (F1-F4, G1). Further, the TRW data were separated by tree-age class and the MGS-NDVI_a_-TRW_a_ relationship was analyzed at the level of age classes. The general idea behind these stratifications was that the tree productivity and hence, the NDVI, is affected by the forest patch size and shows differences even within a patch size depending on the age of the trees as has been reported by Khansaritoreh et al. (2017). Further, it is assumed from previous work that variations in tree productivity are related to variations in climate (Dulamsuren et al., 2010, 2011; Khansaritoreh et al., 2018). Based on this rationale, the hypotheses that are stated in the introduction were tested at the plot level. Mean seasonal MVC NDVI anomalies were extracted for the plot coordinate and the surrounding 3 × 3 pixel neighbourhood in order to ensure that all pixels that contribute to the 20 × 20 m plots are covered. With regard to the hypothesis that grassland NDVI can serve as a proxy for the monitoring of tree productivity (He & Shao, 2006), every forest plot was assigned a corresponding grassland plot. The grassland plots were determined by calculating the shortest distance from the forest plot to the forest border and then measuring a distance of 100 m perpendicular to the forest border outside the forest. Only unmanaged grassland sites were selected.

Statistical analysis was executed with IBM SPSS Statistics 26.0 and R (4.0.3-1). All statistical analysis was carried out at the level of the 15 plots for the 20-year time period. The statistical results based on the calculation of the Pearson correlation coefficient *r* in order to be able not only to evaluate the strength but also the direction of the relations between NDVI and TRW data. The overall significance of the linear models was estimated from an *F* test and the definition of critical *p* values at 99%, 95% and 90% significance level. The results of the statistical analysis were used as input for spatial modelling of annual standardized TRW anomalies for the entire study area. Temporal autocorrelation in the TRW as well as in the NDVI time series observations was tested with the autocorrelation function as described in Venables and Ripley (2011).

## Results

### Landsat time series of maximum growing season NDVI and climate variability

The MGS-NDVI_a_ followed the variations in climate conditions for the studied time period of 1995 to 2014 (Table 1). Higher precipitation during the summer (May–August) was associated to higher NDVI values of the growing season. This correlation could be observed at the plot level for the majority of the 15 forest plots. The highest number of significant relations was found for June precipitation of the same year and seasonal NDVI. Further, the precipitation of the year prior to the NDVI measurement also was correlated with vegetation greenness in terms of NDVI. Here, the significant relations could be observed for the months July and August (Table 1).

**Table 1:** Response of maximum growing season NDVI (MGS-NDVI_a_) to precipitation standardized anomalies of the same year and the previous year (period of analysis: 1995–2014). F = forest-dominated area, G = steppe-dominated area. The first digit defines the forest-patch size, whereas the second digit numbers the single replicas. Plots without second digits represent the summarized classes

	Precipitation							
	Current year				Previous year			
Plot	May	Jun	Jul	Aug	May	Jun	Jul	Aug
F11			Δ					
F12		▲			▲			
F13		Δ						▲
F21		Δ						Δ
F22		▲						
F23							Δ	▲
F31		Δ					Δ	
F32		▲						
F33		▲					▲	▲
F41		Δ	Δ				Δ	
F42		▲					Δ	
F43							Δ	Δ
G11			Δ					Δ
G12	▼	▲						
G13		▲						
F1		Δ						Δ
F2		Δ					Δ	
F3		▲					Δ	
F1-F3		▲					Δ	
F4		Δ					▲	
G1								

▲ significant positive (p < 0.05)

▼ significant negative (p < 0.05)

Δ marginally significant positive (p < 0.1)

**Fig. 4:**
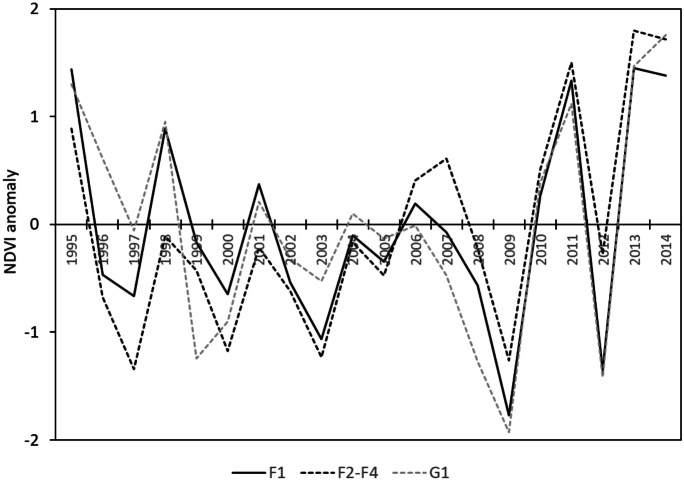
Temporal profile of standardized maximum growing season NDVI anomalies (MGS-NDVI_a_) for the reference period 1995 to 2014 grouped by patch size (F1, F2-F4) and forest-to-grassland ratio (F, G)

The temporal profile of the MGS-NDVI_a_ for the studied time period appeared to reflect these variations quite similarly for the different patch sizes and the separation by grassland- and forest-dominated landscapes (Fig. 4). The general behaviour was similar for all classes with negative standardized anomalies for periods that are linked to years of low summer precipitation (compare with Fig. 2). For single years (e.g. 2012), small forest patches (F1/G1) showed higher anomalies compared with the larger patches (F2 to F4).

## Landsat time series of maximum growing season NDVI and tree ring width

**Fig. 5:**
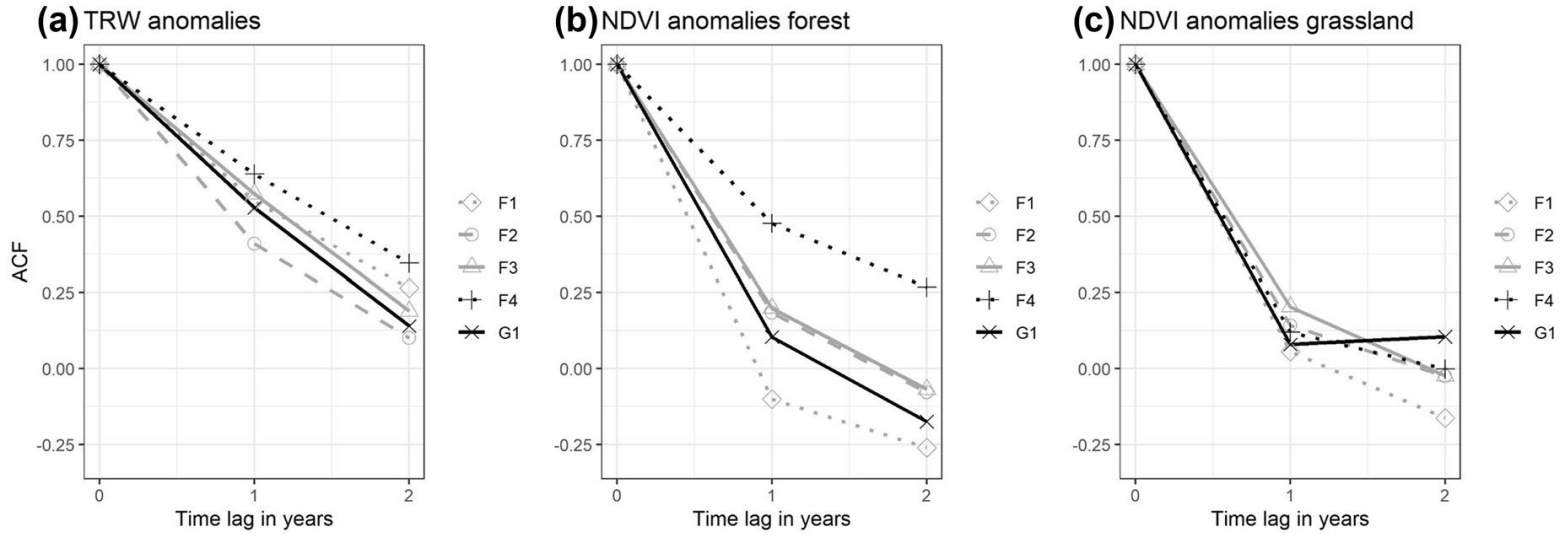
Results of the temporal autocorrelation test. Autocorrelation of annual TRW anomalies **a**, autocorrelation of annual NDVI anomalies over forest **b**, and autocorrelation of annual NDVI anomalies over associated grassland plots **c**. The *X*-axis displays the time lag in years between two observations; the *Y*-axis shows the value of the autocorrelation function. Values below 0.50 indicate no autocorrelation between the observations

The interannual variability of the MGS-NDVI_a_ showed overall significant correlations with TRW_a_ (Table 2). The relation between NDVI and TRW was always positive, i.e. positive standardized anomalies of NDVI were associated with positive anomalies of TRW. Temporal autocorrelation of annual NDVI and TRW observations could be neglected based on the results from the autocorrelation-function test (Fig. 5). Graph (a) in Fig. 5 shows the autocorrelation between TRW of the year under investigation and the previous years (t-1 and t-2). High values indicate a strong effect of the past on the current year. It is obvious that past year’s (t-1) TRW has an effect on productivity of trees in the current year. This is in line with other studies that revealed a spill-over effect of growing conditions of the previous year on current productivity (Babushkina & Belokopytova, 2014; Vaganov et al., 2009). In contrast to the TRW time series, in general, no effect of past NDVI observations on the current year could be found. This is mainly a consequence of the annual aggregation of the NDVI data that eliminates the seasonal cycle and the inherent correlation structure of seasonal data (Forkel et al., 2013) and thus enables a regression analysis on the annual level. Only NDVI of large forest patches (F4) indicated an effect of the past on the current year. This can be due to the stable microclimate of large forest patches that is less effected by climate variability. Annual NDVI values of grassland showed no overall temporal autocorrelation.

**Table 2:** Results of the correlation analysis between standardized maximum growing season NDVI anomalies (MGS-NDVI_a_) and standardized anomalies of the tree-ring width (TRW_a_) for different forest-patch size classes and tree age classes

	Tree age class					
Patch size class/plot	*Y* (≤ 60 years)	*M* (61–100 years)	*O* (101–160 years)	vO (> 160 years)		Mean
F11	-	-	0.33n.s	0.40 +		0.37n.s
F12	-	0.66 + + +	0.49 + +	0.64 + + +		0.60 + + +
F13	-	0.69 + + +	0.78 + + +	0.82 + + +		0.76 + + +
F1	-	0.58 + + +	0.56 + +	0.65 + + +		0.61 + + +
F21	0.41 +	0.48 + +	0.61 + + +	0.63 + + +		0.58 + +
F22	-	0.40 +	0.70 + + +	0.71 + + +		0.69 + + +
F23	-	0.77 + + +	0.85 + + +	0.86 + + +		0.84 + + +
F2	0.53 + +	0.74 + + +	0.82 + + +	0.82 + + +		0.81 + + +
F31	0.67 + + +	0.68 + + +	0.64 + + +	0.73 + + +		0.74 + + +
F32	-	0.27n.s	0.75 + + +	0.59 + + +		0.58 + +
F33	0.52 + +	0.65 + + +	0.53 + +	0.61 + + +		0.60 + + +
F3	0.69 + + +	0.68 + + +	0.77 + + +	0.76 + + +		0.75 + + +
F41	− 0.03n.s	− 0.23n.s	− 0.11n.s	0.03n.s		− 0.08n.s
F42	0.73 + + +	0.73 + + +	0.76 + + +	0.72 + + +		0.75 + + +
F43	0.50 + +	0.55 + +	0.57 + +	0.62 + + +		0.57 + +
F4	0.58 + +	0.70 + + +	0.64 + + +	0.69 + + +		0.66 + + +
G11	0.64 + + +	0.61 + + +	0.68 + + +	0.60 + + +		0.70 + + +
G12	0.66 + + +	0.67 + + +	0.64 + + +	0.47 + +		0.66 + + +
G13	-	− 0.20n.s	0.54 + +	0.55 + +		0.42 +
G1	0.67 + + +	0.54 + +	0.69 + + +	0.63 + + +		0.66 + + +

+  +  + Correlation highly significant (*p* ≤ 0.01), positive

+  + Correlation significant (*p* ≤ 0.05), positive

+ Correlation marginally significant (*p* ≤ 0.1), positive

*n.s* no significant correlation

“-” no tree ring data

Significant correlations (*p* ≤ 0.05) between MGS-NDVI_a_ and TRW_a_ were found for the majority of forest plots over all age classes as well as for the mean of all trees within a plot and therefore also independent of forest-patch size (Table 2). Only two plots (F11, F41) did not show any significant relationships, at neither of the three investigated levels. The highest share of highly significant correlations was found for the age class “old trees” (O). Note that the results for young trees (Y) are less robust for interpretation across plots due to missing data in almost 50% of the plots. The introduction of a 1-year lag in the NDVI-TRW relationship reduced the overall significance of the correlations considerably. Then, significant relations (*p* ≤ 0.05) were only found for small forest patches in grassland-dominated landscapes (G1).

**Fig. 6:**
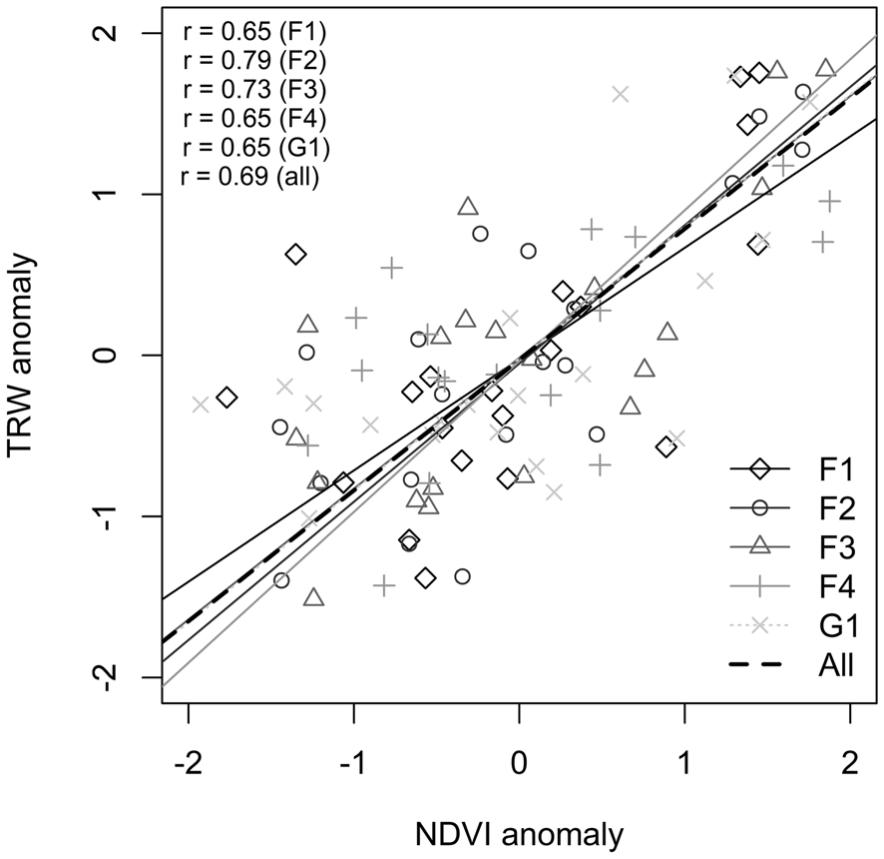
Relationship between standardized maximum-growing season NDVI anomalies (MGS-NDVI_a_) and standardized tree-ring width anomalies (TRW_a_) for different forest-patch size classes (grey lines) and overall correlation for all plot types (black dashed line). Reference period is 1995 to 2014

Based on the significant relations between MGS-NDVI_a_ and TRW_a_, the slope of the linear regression of TRW from NDVI was further investigated (Fig. 6). The underlying assumption that the forest patch size and forest-grassland ratio have an impact on the slope of the regression line was not confirmed. The slopes are not notably different for the different forest-patch size classes.

## Grassland NDVI as a proxy for tree productivity

**Fig. 7:**
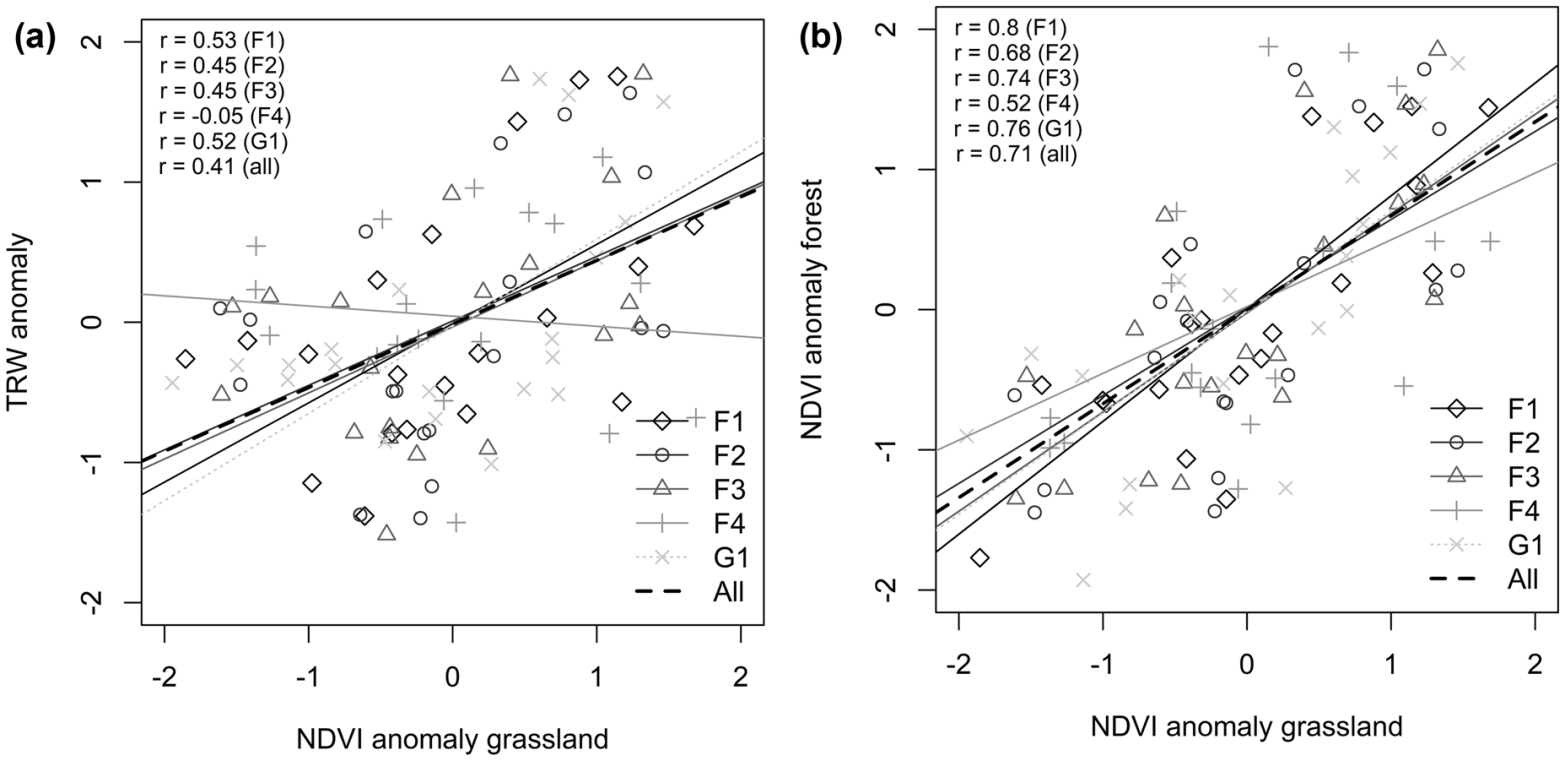
Relationship between standardized maximum-growing season NDVI anomalies (MGS-NDVI_a_) of grassland plots and standardized tree-ring width anomalies (TRW_a_) for corresponding forest-patches **a** and relationship between MGS-NDVI_a_ of forest patch and corresponding grassland plots **b** for different patch size classes (grey lines) and the overall correlation (black dashed line). Reference period is 1995 to 2014

With regard to the hypothesis that steppe-grassland NDVI can be used as a proxy for forest NDVI in the assessment of anomalies and trends in forest productivity, significant correlation between MGS-NDVI_a_ of the steppe and TRW_a_ could be found for all patch-size classes except F4 (Table 3, Fig. 7a). However, the significance levels were generally lower compared with the use of forest NDVI. In many cases, at patch class level and at plot level, no significant relation was observed. The largest forest patches (F4) showed weak correlations and no significance at all. Accordingly, the correlation analysis between grassland and forest MGS-NDVI_a_ revealed highly statistically significant relations (*p* ≤ 0.01) at all forest-patch size classes except for F4 (*p* ≤ 0.05) (Fig. 7b). Here again, the slopes of the linear regression functions for the patch-size classes (F1-F3, G1) are not statistically different, whereas MGS-NDVI_a_ and TWR_a_ of the largest patch size class F4 are completely uncorrelated.

**Table 3:** Results of the correlation analysis between standardized maximum growing season NDVI anomalies (MGS-NDVI_a_) and standardized anomalies of the tree-ring width (TRW_a_) for different forest-patch size classes and tree age classes. NDVI anomalies refer to the corresponding grassland plot at the forest border of the respective patch

	Tree age class				
Patch size class/plot	*Y* (≤ 60 years)	*M* (61–100 years)	*O *(101–160 years)	vO (> 60 years)	Mean
F11	-	-	0.41+	0.47++	0.45++
F12	-	0.48++	0.31n.s	0.49++	0.44+
F13	-	0.44+	0.51++	0.58++	0.51++
F1	-	0.48++	0.47++	0.57+++	0.52++
F21	0.06n.s	0.04n.s	0.16n.s	0.22n.s	0.15n.s
F22	-	0.18n.s	0.48++	0.49++	0.45+
F23	-	0.46++	0.58++	0.59++	0.55++
F2	0.23n.s	0.39n.s	0.53++	0.56++	0.51++
F31	0.61+++	0.66++	0.46++	0.46++	0.58++
F32	-	0.37n.s	0.55++	0.43+	0.43+
F33	0.19n.s	0.39n.s	0.30n.s	0.20n.s	0.28n.s
F3	0.46+	0.43+	0.51++	0.46+	0.48++
F41	− 0.23n.s	− 0.02n.s	− 0.24n.s	− 0.29n.s	− 0.23n.s
F42	0.12n.s	0.09n.s	0.13n.s	0.18n.s	0.12n.s
F43	− 0.15n.s	− 0.16n.s	− 0.01n.s	− 0.13n.s	− 0.13n.s
F4	− 0.15n.s	− 0.08n.s	− 0.13n.s	− 0.18n.s	− 0.14n.s
G11	0.43+	0.42+	0.53++	0.45++	0.50++
G12	0.69+++	0.64+++	0.55++	0.41+	0.61+++
G13	-	− 0.35n.s	0.31n.s	0.37n.s	0.19n.s
G1	0.59+++	0.43+	0.54++	0.47++	0.53 ++

+++ Correlation highly significant (*p* ≤ 0.01), positive

++ Correlation significant (*p* ≤ 0.05), positive

+ Correlation marginally significant (*p* ≤ 0.1), positive

*n.s* no significant correlation

“-” no tree ring data

**Fig. 8:**
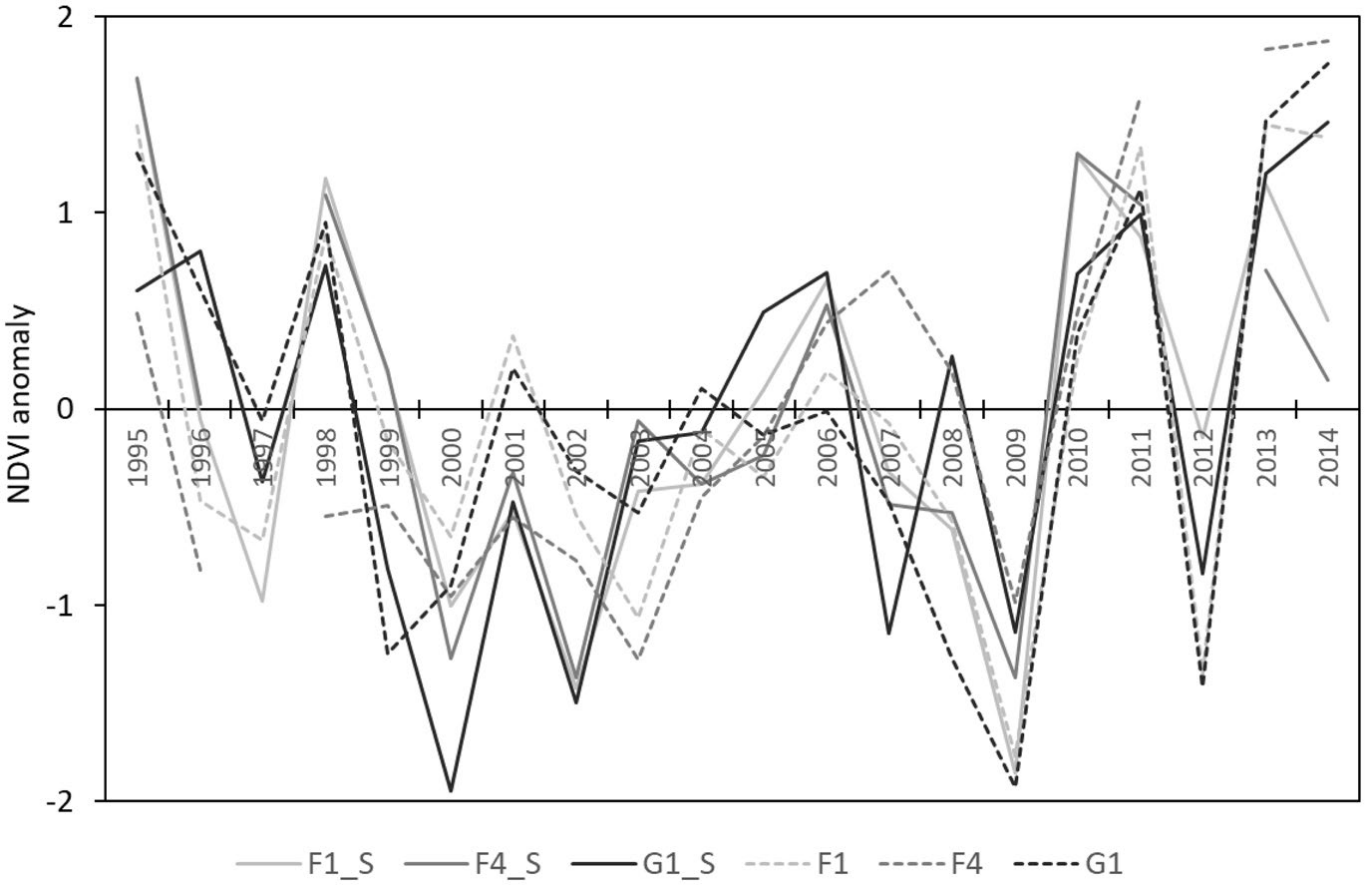
Standardized maximum-growing season NDVI anomalies (MGS-NDVI_a_) for forest-patch size classes (F1, F4, G1) compared with corresponding steppe plots (F1_S, F4_S, G1_S) (reference period 1995 to 2014)

**Fig. 9:**
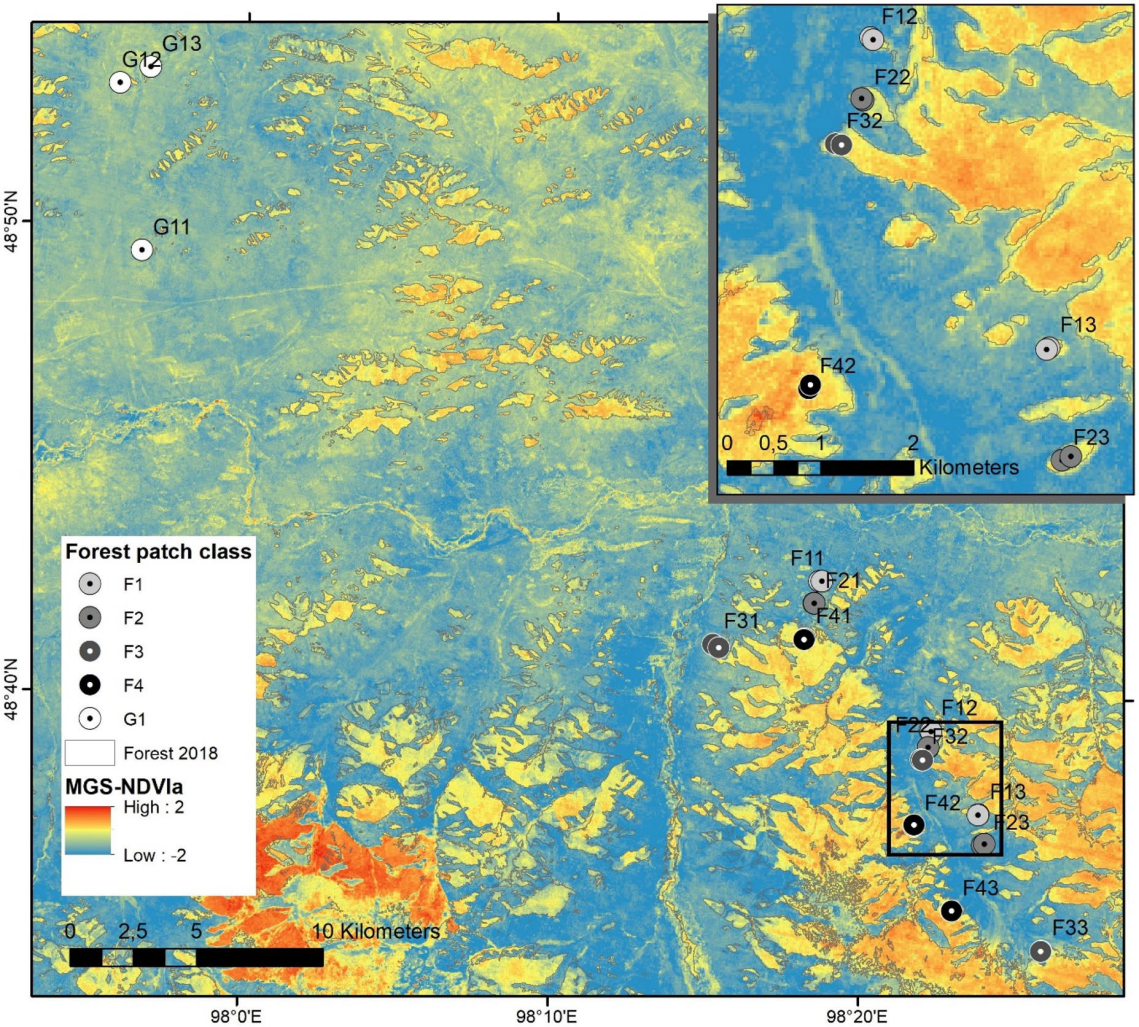
Spatial patterns of standardized anomalies of maximum growing season NDVI (MGS-NDVI_a_) for the year 2002. Outline shows the extend of forest patches, dots illustrate the location of the sample plots for the different forest patch classes

A closer look at the temporal signatures of grassland and forest NDVI anomalies (Fig. 8) confirms the generally uniform behaviour of the two time series for small forest patches (F1, G1). However, it can also be seen that the general response of forest NDVI in some years is different from grassland NDVI, especially in large forest patches and their surroundings (F4). The large discrepancies between forest and steppe NDVI anomalies become obvious, especially in drought years, which is exemplarily shown in Fig. 9.

## Discussion

We used a compilation of dendrochronological records from 15 plots of Siberian larch in the southern boreal forests of Mongolia together with a dense time series of Landsat satellite images for 20 years to investigate the relations between annual radial stem increment and maximum NDVI composites of the growing season. The study concept followed the evidence from previous literature about the general similarity of temporal patterns from NDVI as an indicator of vegetation greenness and tree-ring width (TRW) as a proxy of biomass accumulation. Hence, both signals represent the vegetation vitality and can indicate variations in forest productivity (Kaufmann et al., 2004; D'Arrigo et al., 2000). Our results confirmed the correlations of NDVI to annual variations in climate during the growing season and thus TRW for the mountainous forests in the study area.

The seasonal maximum of vegetation greening in terms of NDVI is closely linked to the precipitation in the summertime (most frequently in June) of the corresponding year of observation. Late summer and spring rainfall did not affect the maximum growing season NDVI. This is in accordance with other studies (Bumann, 2017; Kaufmann et al., 2004) that also reported a close relation of growing-season precipitation to the maximum of NDVI. On the other hand, He and Shao (2006) did not reveal any significant relations for Qilian juniper stands in central China. In comparison with the NDVI-precipitation relationship, Khansaritoreh et al. (2017) pointed out that the TRW-precipitation relationship is more correlated by applying a 1-year time lag between summer precipitation and TRW. Nevertheless, like other authors, they found significant precipitation-TRW relationships for both the current and previous year summer months (Bumann, 2017; Khansaritoreh et al., 2017). In accordance with these findings, we could show both a relation between NDVI and precipitation of the same year as well as a weaker but still significant relation for NDVI and previous year July precipitation.

In opposite to our finding of significant correlation of data from the same year, Wang et al. (2004) reported a 1-year lag in the correlation between NDVI and TRW for oak forests in North America. The maximum growing season NDVI was found to be a valuable indicator of tree productivity, whereas the sizes of the forest patches and the landscape type (grassland vs. forest dominated landscapes) did not have an impact on the NDVI-TRW relationship. The general relationship between NDVI and TRW was reported by other authors, too (Bumann, 2017; Coops et al., 1999; He & Shao, 2006). Further, Khansaritoreh et al. (2017) found that TRW in small forests compared to larger forests correlated more with climatic variability, suggesting that small stands show a higher variability also in NDVI. Indeed, small stands showed higher amplitudes of NDVI over the observed time period in both forest-dominated and grassland-dominated forest-steppe areas (Fig. 4). Dulamsuren et al. (2019) reported that the humus content of the organic layer, which improves the moisture availability, increased simultaneously with forest stand size. In addition, Klinge et al. (2021) have shown that permafrost is promoted by a thick organic layer and permafrost is mostly absent in small forest stands. The authors stated that permafrost supports soil-moisture conditions, which influences tree growth in the forest-steppe. This geoecological factor decreases drought stress in large forest stands, whereas small fragmented forest patches are subjected more to climate variability. Nevertheless, this increased climate sensitivity of TRW and NDVI in small forest stands did not obliterate the general correlation between the two variables across all studied forest stands.

Due to data quality issues and availability in the early years of Landsat 5 TM, the time series in our study had to be reduced from 31 to 20 years of overlapping dendrochronological records and annual NDVI composites. This is in line with the majority of comparable studies that investigated the sensitivity of NDVI time series to tree productivity based on dendrochronology (D'Arrigo et al., 2000; Bunn et al., 2013; Wang et al., 2004; Vicente-Serrano et al., 2016; Kaufmann et al., 2004; Brehaut & Danby, 2018). The major drawback of the Landsat-based studies is that they were solely based on single acquisitions that represent a full growing season (Coops et al., 1999) or on data from a single sensor, e.g. Landsat 5 TM (Bumann, 2017). This constraint and also the usage of pre-composited products limited the proper delineation of the maximum of growing season NDVI in the earlier studies. Kaufmann et al. (2004) underlined that only the summer peak in NDVI represents the physiological status of the forest stand. Thus, the approach in the present study used the full archive of Landsat data for the delineation of the seasonal maximum NDVI as a proxy for tree productivity. The highly significant results of the correlation analysis confirmed the adequacy of this approach. However, the mentioned issues in the MVC processing for the first 10 years of the time series also reveal the still existing challenges for long time series of satellite data. Bunn et al. (2013) pointed to the fact that despite the major progress in satellite-time series availability and processing, the major restriction of satellite-time series for monitoring tree physiology parameters still is the relatively short record compared with the variation of tree growth at decadal or even multi-decadal time scales. Despite this fact, the present study underlines the indisputable value of high-resolution dense time series for monitoring and retrospective modelling of tree productivity.

A major issue in the NDVI-TRW analysis in highly fragmented forest landscapes is that the forest patch size cannot always be covered and depicted accurately by the spatial resolution of the available satellite-time series. This is why in previous studies, grassland NDVI time series have been used as a proxy to monitor temporal variations in tree productivity (Bumann, 2017; He & Shao, 2006; Wang et al., 2004). This principle builds on the assumption that forests and grasslands grow under similar climatic conditions and presumes a similar response of different vegetation-cover types on variations in those climate conditions (He & Shao, 2006). In this case, forest NDVI can be replaced by grassland NDVI in modelling productivity. In our study, we generally confirmed the overall significant relation between grassland and forest NDVI over the investigated time period. However, in some years, the overall spatial patterns of NDVI and thus TRW standardized anomalies show opposed responses of grassland and forest patches to climate conditions. For example, for the year 2002, we observed strong negative anomalies for steppe-grassland areas whereas the forest areas showed no deviation to the long-term mean (Fig. 9). This year was at the end of a long, severe drought with very low summer rainfall in the Khangai Mountains (compare Fig. 2). This difference is the result of the different life strategies of late-successional forests and grasslands in terms of stomatal regulation. Moreover, large forest patches show a higher resilience to climate extremes compared to small, highly fragmented forest sites. For the specific site conditions in our study area, this can be linked to various environmental factors where the most obvious one is the strong association of forest occurrence with topography. Forest patches are mainly present on north-facing slopes where evapotranspiration is reduced compared to south-facing slopes, resulting in higher soil water availability and relative humidity that support tree growth (Klinge et al., 2018). During drought periods, soils in the forested areas are favoured by canopy shading and are able to maintain necessary hydrological conditions over longer periods. The higher resilience of large forest patches compared to small patches in the study area is also associated to the permafrost distribution that modifies the soil hydrological regime in larger, closed forest stands (Klinge et al., 2018, 2021). Here, seasonal ice above the permafrost layer can compensate the deficiency in drought years by accumulating soil water in moist years and releasing water to the tree roots in dry and warm periods (Sugimoto et al., 2002). Summarizing, the analysis of the grassland NDVI-TRW relationship shows a potential for monitoring tree productivity in highly fragmented grassland-steppe-forest landscapes. It also reveals that this relationship is not uniform but shows spatial and temporal inconsistencies that are a function of complex, site-specific ecological parameters and processes. Thereby, the availability of higher spatial resolution satellite time series as exemplified in this study is a prerequisite to separate those effects.

## Conclusions

Seasonal composites of NDVI data from a 20-year multi-sensor Landsat time series were tested for correlations with dendrochronological tree ring growth of Siberian larch in the southern boreal forests of northern Mongolia. The analysis concept was based on the hypothesis that the general vegetation greenness is correlated with ecosystem productivity and thus productivity can be captured by satellite-based vegetation indices like the NDVI. Our results confirmed the general ability of NDVI time series to capture anomalies in forest productivity. We could also demonstrate that the nature of the relation between the NDVI signal and tree ring width is comparable for different forest types (e.g. age, patch size, forest-steppe ratio). However, our analysis revealed that the NDVI of grasslands is less suitable as a proxy to monitor forest productivity due to the different response patterns of grasslands and forests to climate conditions. Our study should therefore contribute to a better understanding of long-term dynamics in forest-steppe ecosystems and their relation to climate change. Overall, the observed patterns and relations between satellite data time series and dendrochronological records form the basis for a retrospective modelling of tree ring growth over larger areas and thus may provide a key parameter for global climate change and terrestrial carbon cycle studies as well as for the calibration of global carbon models.

## Data Availability

This study used Landsat data that were publicly available from U.S. Geological Survey (USGS) Earth Resources Observation and Science (EROS) Center (https://earthexplorer.usgs.gov/).
